# Facilitating equal access to primary care for all: work experiences of health mediators in a primary health care model programme in Hungary

**DOI:** 10.1186/s12875-020-01281-z

**Published:** 2020-10-17

**Authors:** Cintia Katona, Éva Gutási, Magor Papp, Orsolya Varga, Karolina Kósa

**Affiliations:** 1 Department of Behavioural Sciences, Faculty of Medicine, University of Debrecen, Debrecen, Hungary; 2 Health Development Center, Semmelweis University, Budapest, Hungary; 3 Department of Public Health and Epidemiology, Faculty of Medicine, University of Debrecen, Debrecen, Hungary

**Keywords:** Health mediator, Structured interviews, Primary health care, Group practices

## Abstract

**Background:**

A Primary Care Model Programme was implemented in Hungary between 2013 and 2017 in order to increase access of disadvantaged population groups to primary care and to offer new preventive services for all clients. In a country with single-handed practices, four group practices or GP clusters were created in the Programme. Six GPs comprised one cluster who together employed nonmedical health professionals and nonprofessional health mediators, the latter recruited from the serviced communities, many of them of Roma ethnicity. Health mediators were tasked by improving access of the local communities – including its vulnerable Roma members – to existing and new services. Health mediators were interviewed about their work experiences, motivation, and overall opinion as members of the clusters as part of the Programme evaluation.

**Methods:**

As part of the Programme evaluation, structured interviews were conducted with all 40 health mediators employed at the time in the Programme. Interviews were transcribed and content analysis was carried out.

**Results:**

Three themes emerged from the transcripts. The first focused on the health mediators’ personal characteristics such as motivation to join the Programme, the way their job increased their self-esteem, social status and health consciousness. Domains of the second theme of their work included importance of on-the-job training and of their insider knowledge of local communities, as well as their pride to have become members of the primary care team. The third theme covered overall functioning of the Programme of which they had mostly positive opinions, notwithstanding some criticism regarding procurement.

**Conclusions:**

Health mediators had earlier worked in various European countries specifically to improve access of Roma ethnic groups to health services but the Hungarian Model Programme was globally the first in which health mediators as non-professional workers became equal members of the primary care team as employees. Their contribution and overwhelmingly positive experiences, along with their useful insights for improvement call for the establishment and funding of health mediator positions in primary care especially in areas with large numbers of disadvantaged Roma populations.

## Background

Roma people comprise the largest ethnic minority in Europe and in the European Union (EU) who in most countries have been facing prejudice and social exclusion. The EU has repeatedly called for better integration of Roma, and developed an EU Framework for national Roma integration strategies in 2011 that identified education, employment, housing and healthcare as key areas of integration [1]. Healthcare was also identified by the Council of Europe as a critical area that was addressed in a Council recommendation on better access to health care for Roma and Travellers in which, among others, governments of member states were requested to ensure geographically accessible and affordable health care [2].

Utilization of primary health care services, particularly preventive services, is one of the means by which Roma health could be improved as it has been shown to be consistently lagging behind the health of majority groups according to various indicators [3–5].

However, access to and utilization of primary health care services has been laden with problems among vulnerable Roma population groups, such as lack of health insurance and/or necessary documentation, financial constraints, discriminatory attitudes experienced from health care staff, lack of trust in health care providers, and difficulties in handling the complexities of the health care system that result in the underutilization of services [5–8].

In order to bridge the gap between healthcare providers and vulnerable Roma groups, health mediation was introduced and tested in several countries with positive experiences [9]. Health mediation stems from the concept of mediation that aims at facilitating communication and understanding between parties in disputes in order for such disputes to be settled amicably. Mediation has been widely used in business and law as an alternative method of dispute resolution leading to a concrete agreement [10].

The history of health mediation goes back to the 1990s in Romania where mediators of Roma ethnicity had been recruited to help resolve a stalemate that emerged during a vaccination campaign. The model for training and employing Roma health mediators (RHM) was further developed in the framework of the Roma Health Project of the Open Society Foundation in the beginning of the 2000s. Most countries that participated in the Decade of Roma Inclusion running from 2005 to 2015 developed such health mediator Programmes, and their experiences were published in detailed reports [11, 12].

The present paper describes the experiences of health mediators employed in a large-scale primary health care model Programme (called “Primary Care Development Model Programme”) in Hungary which was created in the framework of a bilateral agreement between the governments of Switzerland and Hungary, and was funded by the Swiss-Hungarian Cooperation [13]. The Programme had two major aims: to remodel primary health care with a focus on preventive services, and to reduce health inequalities between vulnerable Roma and the majority population. Relating to the latter aim, the Programme had a special workgroup dedicated to Roma health issues. The Programme was implemented in four disadvantaged areas of the country with sizable Roma minorities. Health mediators recruited from the serviced vulnerable communities had been employed as members of the primary care teams as described in detail in the Programme’s Operations Manual [14]. The work responsibilities and contribution of health mediators in this Programme were described in detail elsewhere [15]. Briefly, four groups of general practitioners were established as primary care group practices (called “GP clusters”). Each group practice consisted of six general practitioners who received funding to employ a range of ancillary health professionals such as public health specialist, dietitian, physiotherapist, health psychologist, and non-professional health mediators [16]; and offered new, preventive services not available from other GPs [17]. The Programme started in 2013 and ended in 2017.

Forty-eight health mediators (12 per GP cluster) were employed in part-time positions at the beginning of the Programme in 2013 though the number of health mediators changed through the duration of the Programme as described in detail elsewhere [18]. All mediators had been recruited from the local communities as non-professional health workers; most of them identified themselves as Roma. Mediators – in accordance with the Rules of Procedures of the Programme [14] – provided help to healthcare professionals in the GP clusters in terms of logistics and organization, under the supervision of the public health coordinator.

The main task of the health mediators was mediating between professionals in primary care and the local population, with special attention to those living in disadvantaged socio-economic conditions. In particular, they were responsible for increasing attendance at the health status assessment, a general screening for all adults of the GPs based on a specified screening protocol [15]. Adult patients of the GPs were invited by letter for the assessment, and health mediators had to get in touch with those who did not show up and persuade these people to go. Health status assessment was comprised of a questionnaire survey and health examination; mediators were tasked to assist in the questionnaire survey for those who requested it because of visual or comprehension problems. Mediators also participated in the organization and in implementation of various lifestyle counselling and health promoting Programmes provided to groups and the local communities. They also provided help – if requested – to the health visitors who were responsible for the monthly mother-baby clubs, attended by mothers and their babies as well as expectant mothers. Health mediators attended to the babies during the clubs so that the parents (mostly mothers) could pay attention to the presentations and discussions.

Vocational training of 800 h of duration in assistant nursing was provided in the first year of employment to all those health mediators who had the appropriate educational qualification to enter training, who did not yet have any health-related vocational qualification and who volunteered to complete the training. 3-day training in health mediation was provided to all health mediators in the first and third year of employment in the Programme, also fully paid for and completed during work hours. All expenses related to both the vocational and mediator trainings were paid for which were completed during work hours. A number of 1- or 2-day short courses of continuing education were completed by mediators between 2013 and 2017. Health mediators were coordinated and supervised by the so-called public health coordinator, the non-medical leader of the GP cluster.

As part of the evaluation of the Programme, all health mediators employed in the GP clusters were interviewed about their work experiences in the last half year of the Programme according to its original schedule (the Programme was originally planned to finish in June 2016 but became extended until May 2017). The present paper describes the analysis and findings of these interviews uncovering the experiences of health mediators while employed in the model Programme.

## Methods

### Study design

A qualitative study was carried out based on individual interviews as described in the literature [19] with health mediators of the GP clusters of the Primary Care Development Model Programme. Structured interviews in relation to 12 topics were conducted with mediators. Health mediators had the opportunity to talk about any other topic at the end of the interviews. Ethics approval was received from the relevant national body as detailed in the section “Ethics approval”. All interviewees were briefed about the aims of the research project and the interviews in a meeting with management in January 2016, and oral consent was obtained from each health mediator before the interviews.

### Participants

All forty health mediators employed in January 2016 in the four GP clusters of the Primary Care Development Model Programme were briefed about the research and invited to be interviewed voluntarily, to which all agreed. In order to avoid potential influence from their supervisors (the public health coordinators), interviews took place after work hours, and the supervisors did not know who was interviewed. The interviews were conducted in a public workspace (waiting room, community room) of the GP cluster to which the mediators had access after their work hours and outside of service hours. Interviews were conducted by the first and second authors who were members of the special workgroup dedicated to Roma health issues and who had personally known the mediators but were neither their co-workers nor their supervisors; and two junior researchers who had been in contact with the mediators in relation to other projects but were not employed by the Programme. The aim of the research and methodological details of conducting the interviews were extensively discussed with all interviewers in order to increase methodological homogeneity. The interviews were conducted between February and May 2016. Average duration of the interviews was 23 min; the shortest one took 9, while the longest one 47 min. All interviews were conducted in Hungarian, recorded without names, and transcribed into Microsoft Word documents. The audio files were deleted after transcription. Numerical codes were used to identify participants in the interviews and transcripts. The transcripts had a total length of 104,977 words on a total of 241 pages.

### Structure of the interview

Interviews were conducted using 12 pre-defined questions arranged in four groups. The first group of questions asked about health mediators’ reasons to join the Programme; potential changes their employment brought in their lives and status in the family and community, as well as their ethnic origin and the role their ethnicity might have played in being employed in the Programme. The second group of questions related to their most important job responsibilities, typical work-related conflicts and stress situations in relation to co-workers and clients, means to deal with such situations, and their own behaviour and being a role model for others. The third group of questions asked their opinion about the model Programme, its strengths and weaknesses, cooperation with other employees of their GP cluster and their supervisor, the public health coordinator. The fourth group of questions concerned the interviewees’ image of the future, and any other opinion or comment they thought important but was not addressed during previous questions. The end of the Programme was planned for June 2016 at the time of the interviews that was later extended to May 2017.

### Data analysis

Qualitative content analysis was carried out according to the literature [19]. After multiple readings of the transcripts by the first and second authors, codes were identified and discussed until agreement was reached. Computer-assisted analysis was also tested by NVivo 12 qualitative data analysis software [20] but its usefulness was limited due to language barriers since analytical options depend on the text content language. Eight analytical subthemes emerged from the codes that were organized into three major themes. The first and second authors independently coded the interviews, subsequently extracting subthemes and themes, and the other three authors supervised the process. Iterations continued among all authors until they reached agreement.

## Results

All 40 health mediators employed in the Programme at the time of the interviews in the 4 GPs practice clusters (7 in GPC1, and 11 in the other 3 GP clusters [GPC2-GPC4]) consented to be interviewed. Their demographic characteristics are shown in Table 1.

**Table 1:** Characteristics of the interviewees

Characteristics of the interviewees	January 2016
Number of health mediators in the Programme (persons)	40
Number of interviewees (persons)	40
Women (%)	93%
Self-identified Roma (%)	73%
High school graduates with maturity exam (GCSE) (%)	18%
Vocational training in healthcare before joining the Programme	8%
On-the-job vocational training (%)	38%
Unemployed before entry into the Programme (%)	63%
Part-time position (%)	80%
Full-time position (%)	20%

**Fig. 1:**
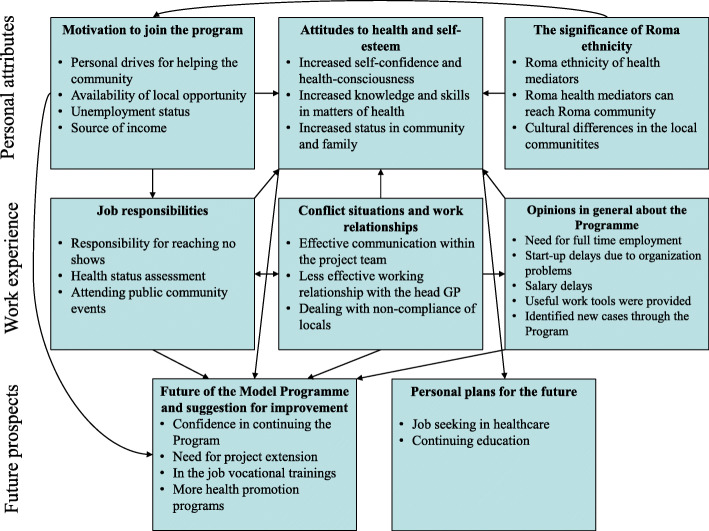
Model of work experiences of health mediators in a primary care model Programme

Three major themes emerged from the content analysis and coding of the interviews. The first related to the personal attributes of health mediators, including their motivation to join the Programme, their daily life, and the significance of their ethnic identity. The second theme focused on their work-related experiences: relationships with co-workers and clients, their work responsibilities, and their opinion about the operation of the Programme. The third theme was related to their views on the future of the Programme and their personal plans in the future. The graphical summary of findings is shown in Fig. 1 presenting an overview of the model of work experiences including themes, subthemes with their background nodes and also their relationships.

### Theme 1: Personal attributes

#### Motivation to join the Programme

Two-third, 25 out of the 40 health mediators had been unemployed before joining the Model Programme even though all of them would have wanted to work. One who experienced a long period of unemployment put it this way:

*“…it is not the only purpose of my existence to cook and bake at home like a domestic servant. I do want to work in a job, and then also do the housework on top of that.” (GPC4/11.)*

Nineteen persons emphasized that their knowledge of the communities and the possibility of helping people was a motivating factor for them.

*“First, I like to interact with people, and those who don’t go to the doctor or don’t want to go, I can reach out to them.” (GPC1/2.)*

Ten persons mentioned that the local availability of the job and half-time employment allowed flexibility that they needed to also take care of their children.

*“For me, it was very good that, I have two children, I need to work in my village, I didn’t have to go to another place.” (GPC4/1.)*

Interestingly, only a few of the interviewees mentioned earning income as a primary motivating factor.

#### Attitudes to health and self-esteem

All respondents stated that their lives changed for the better by joining the Programme. Half of them said that their self-esteem increased since working here. They said they felt greater self-confidence and health-consciousness compared to the beginning of the Model Programme. More than two-third of the respondents considered themselves health-conscious:

*“… my life changed a lot, I meet a lot of people, and I, even myself, became more health-conscious since I am working here.” (GPC4/6.)*

In one case, confidence even seemed a little stretched:

*“…you know, it’s like a synonym, healthcare and I, we belong together.” (GPC1/4.)*

Increased knowledge in matters of health, disease and healthcare procedures was mentioned by several mediators who completed a vocational training in assistant health nurse training provided in the Programme (20 persons).

*“I learned new fields in healthcare … I have knowledge and I know things that I did not know before…” (GPC1/6.)*

One interviewee gave a concrete example of her skills that were commended the emergency crew:

*“After I finished vocational training, it happened that I came across somebody on the street who was involved in an accident and he was bleeding and I quickly put together a pressure bandage and when the paramedics arrived, they asked who put it on, and they said good job”. (GPC3/10.)*

In terms of their own health, they experienced improvements:

*“My health has improved, my spirit is better, and I feel well among people. That fact that we help others makes me stronger too.” (GPC1/4.)*

They attributed a major importance to nutrition. Several had participated in nutrition counselling sessions provided by their dietitian co-workers and changed their own families’ diets accordingly. One of them reported to have lost 17 kg during the Programme, personally helped by her dietitian co-worker. They considered setting an example and being authentic of outstanding importance.

*“I have made up my mind that things cannot go like this at the age of forty that I cannot be locked into my own body, and at this point, I started a lifestyle change, so it was already worth it because of that.” (GPC 2/8.)*

Several respondents reported that the local population’s trust in them had also increased, and that they had increased authority within the community. One health mediator explicitly said that she was proud to work along a doctor, and that it had an influence on how she and her family were seen by the community.

*“We looked at the doctors as if they were gods, we respect them and all. And then we could also enter into their sphere.” (GPC2/1.)*

Some mediators expressed an understanding that increased status meant greater responsibility for being healthy and setting an example in order to be a role model to their peers and their children:

*“I am not just a health mediator, but a doctor among the children. And the generations who have known me and I’ve grown up with, they look up to me. I am proud of this and the others are also proud of me.” (GPC2/5.)*

Some of them felt that people are proud of them, and they respect their person and their work. Their role within the family was also transformed. Their reputation improved in their families; family members supported their work, and their families overwhelmingly (39 out of 40) evaluated their work in a positive light. Some of them reported that they had become more talkative and self-assertive at home as well.

*“I definitely speak more, that’s for sure, and I can explain things better also for the children at home. And then [the child] says, ‘Mum, you are not writing a report now, you don’t have to put it in such a fancy way.” (GPC2/10.)*

#### The significance of Roma ethnicity

Roma (or any) ethnicity was no requirement at recruitment but the job description stated that working with local Roma communities would be part of the job. 72% (29 out of 40) respondents identified themselves as being Roma, four persons identified as Hungarian, three stated to have a dual (Roma and Hungarian) ethnic identity, and four respondents were uncertain. One of the latter put it this way:

*“The Roma or Gypsies are a separate ethnic group. But since we do not speak their language or follow their traditions, or do anything else that could be described as Roma, but I am not Hungarian either, at least not that kind of [majority] Hungarian, but … however, I also do not, I would not consider myself to be Roma either.” (GPC2/4.)*

This respondent said she believed she had been chosen for the position because her family occupied a “prestigious” place in the community, and the GP and the locals had all known them and considered them to be reliable. Those who identified as Roma said that they considered their ethnicity important in their work, because Roma health mediators are more at ease in their communication with the local population, many of whom they personally know anyway.

*“…Roma people are not easily accessible, and this had to be bridged between the doctor and the Roma, this connection.” (GPC2/1.)*

They had been well aware of the cultural differences in the local communities:

*“If you want to help the Roma to catch up, then you do have to work with the problematic families. And you have to help them so that they can keep their pace in terms of hygiene and nutrition.” (GPC2/1.)*

63% of the respondents considered it important to employ Roma persons in healthcare in general, and all mediators agreed that the employment of Roma in the intervention area of the Model Programme was indispensable due to their intimate knowledge and acceptance of Roma culture and behaviour.

*“Someone once said that they were going to examine her neck. Because we told them it was a cervical screening [in Hungarian: cervix = neck], so she said, what do they want to look at on my neck?” (GPC2/9.)*

### Theme 2: Work experience

#### Job responsibilities

Health mediators were responsible for getting in touch with those who were invited by letter but did not show up at the health status assessment:

*“It is difficult to convince some people to come [to go to the health status assessment]” (GPC1/2.)*

They had to get in touch in person with the no-shows, and this gave rise to conflict situations that they made great efforts to resolve (see below). However, others from the community were willing to talk to the health mediators but not with their doctor:

*“…the fact that we moved a lot of people who had not seen their doctor, or been to any screenings for years. There were a lot of people coming in to see us who were, let’s say, aware of having some kind of an illness, but not its consequences.” (GPC4/5.)*

In addition to recruiting for health status assessment, they also provided help – in accordance with the Rules of Procedures of the Programme – to healthcare professionals in the GP clusters in terms of logistics and organization, under the supervision of the public health coordinator.

*“…we take blood pressure in the waiting room, not in the GP’s office. But we also go out to the village to grab those who did not show up [for the health status assessment], because this is our main task.” (GPC3/7.)*

They also provided help – if requested – to the health visitors who were responsible for the monthly mother-baby clubs, attended by mothers and their babies as well as expectant mothers. Health mediators attended to the babies during the clubs so that the parents (mostly mothers) could pay attention to the presentations and discussions. They participated not only in the organization but also in the implementation of various lifestyle counselling and health promoting Programmes provided to local groups and communities.

*“Afterall, they [local community members] got to know us, because we were present at all those events [for the communities] when anybody could come, and if they [the GP cluster] organized a screening for breast or lung cancer, we were there and we helped everyone.” (GPC4/8.)*

#### Conflict situations and work relationships

The main conflict situation emerged with local residents who were invited but did not show up at health status assessment. In such cases, mediators received the address of the person, had to get in touch with them in person, and convince the client to come to the assessment. One mediator illustrated the difficulties with one of her own encounters:

*“I went to an old man’s house, rang the bell for a long time, and after a while, the man shouted out through the window: ‘I am not at home!” (GPC1/7.)*

Two of the mediators reported that they had cases where the person considered this invitation as harassment; in such cases they asked for the help of the physician.

*“…[some] did not want to come [to health status assessment] at all. And we had to go next week and the week after, and they took this as harassment…” (GPC2/11.)*

Health mediators working with paediatricians frequently encountered problems when checking for lice. However, this has been resolved through their personal knowledge of the mothers and understanding communication. Intimate knowledge of the community and humour worked even in those few cases when the health mediator was male:

*“…when we came to check the kids during the examination, and they would always say, look, here’s your mother coming again to check for the lice and everything. They teased him [the male health mediator] with this. But in the end, they got used to that. And they also really like it, they accept it, he’s so proud of me now, they’re so proud of me, I’ll say it like that, and that makes me feel good. Especially now that we have done this training ([on-the-job vocational training], it’s even better.” (GPC2/9.)*

Health mediators mostly worked in pairs. Stressful situations that they encountered were typically discussed on the same day with their immediate co-workers, immediate supervisor (the public health coordinator), or with family members. Mediators working with paediatricians consulted the health visitors in relation to problematic cases. Health mediators working in the same GP cluster had a monthly meeting with their supervisor, the public health coordinator during which conflicts could be discussed, and the experiences of mediators could be exchanged.

“If the recruitment didn’t go so well somewhere, and, for example, for those living in B* it always went better, then they would always say that this is the way they do it and we should try that as well. And, then, sometimes it actually worked like that. Or there were times when our way went better, and then we helped them.” (GPC2/3.)

They found work-related administration adequate, and thought they received necessary information for their work, as well as recognition both in terms of their work and personally as well. The majority of the respondents (38 persons) mentioned that they had a good relationship with the public health coordinators (their supervisors), found them helpful, reliable, flexible and empathetic. They thought their supervisors, the public health coordinators treated them as equal partners though all the coordinators had been graduates and tended to be younger than most of the health mediators (hence the reference to them as ‘kids’).

*“I think they are very fine kids.” (GPC4/9.)*

*“They are helpful, because whenever there’s a problem or anything, they help with everything.” (GPC1/6.)*

However, 11 mediators thought there was too little communication with the head GP, the director of the entire GP cluster. 4 respondents specifically mentioned that they received the head GP’s approval late for some of their work-related requests. Sometimes they received tasks that were difficult to complete because of a tight deadline. For example, they only received the list of those who they had to invite personally for the health status assessment a few days before the due date. They all received the same-size of white gowns that did not fit all of them. There were instances when the lack of proper space became an issue when the mediators tried to help those who could not fill the health questionnaire themselves.

*“They [management] can’t see it that we are put in the hallway for years, I guess just put out there. This was a big grievance for us, because when there was a lot of other patients around, we didn’t really like to read out the questions of a psychological test for everyone else to hear. We had to find somewhere a more private place so the other patients wouldn’t hear us.” (GPC4/10.)*

#### Opinions in general about the Programme

Respondents highlighted several strengths of the Programme. They thought it was well-organised, they appreciated the teamwork and the equal treatment of workers, including the non-medical health professionals such as dietitian, physiotherapist, psychologist. They thought that the involvement of local partners such as the local governments and schools was important, and thought it useful that many Programmes were organized in a wide range of settings like kindergartens, schools, retirement homes.

*“So, we help to prepare health education lectures, and the organization of Programmes. And it is very important, when we go to the kindergarten and school and the children listen to the presentations, and we help [the health professionals] to deliver the Programme.” (GPC4/7.)*

They thought the health status assessment for which all patients of the participating GPs were invited as very important. They cited a number of examples when patients with high blood pressure and diabetes were identified during health status assessment and sent for further examination in time; clients were reached who had not been seen by doctors for years; many persons got encouragement to participate in various extra services not previously provided by the GPs, and the number of children with lice decreased.

*“Well, many people turned out to have diseases that they had for a long time but they did not care about. We found a lot of diabetics, those with narrowed [blood] vessels, many-many with high blood pressure.” (GPC3/5.)*

*“I think the [health status] assessment was the best because people could learn about themselves, their health, and many problems came to light. We found many with high blood pressure.” (GPC2/11.)*

They had been aware of the fact that participation at the health status assessment at the time of the interviews had already been high. All health mediators mentioned in some form their own contribution towards this result, attributing an important part to themselves.

*“The strength about this, as I said, is that we really reach those people and they go to the doctors, even though they haven’t seen them in years.” (GPC2/1.)*

Some put a high emphasis on the mediators’ work:

*“I think the strength of this Programme would be our work. We are the engines of this whole thing, that’s why I said that there is a need for us.” (GPC1/6.)*

Regarding conditions of their own work, they specifically mentioned that bicycles provided to them in the Fall of 2015 greatly helped their work, making their movement in the community much faster and freeing more time for the clients.

*“Receiving the bikes was great because we all use it. Not only in our village but going from one village to the other.” (GPC2/11.)*

They thought their work eased the burden on the doctors and nurses; the community became more open toward doctors, and the Programme set an example for other regions as well.

*“Well, I think they became more open towards doctors, they come more often. They now believe more that it [going to the doctor] makes sense. They loved the [health status] assessment, many women came for that. So they are more open, and they think if they have a problem they are in good hands.” (GPC2/10.)*

Regarding shortcomings of the Programme, they mentioned quite a few. Tools and equipment were not available at the beginning of the Programme, and those ordered usually arrived after a very long time (e.g. bicycles and tablets) due to mandatory public procurement. Contracting of the health mediators was delayed, employment certificates for various purposes issued by the Programme’s management were received slow and/or late (e.g. one of the health mediators’ child did not receive a scholarship as consequence). Their salaries sometimes arrived late, and twelve mediators had problems with the amount of family tax allowances but four of them could not decide if this was due to miscalculation by management or not; and eight persons had no insight about this at all.

*“Shortcoming? I would say missing tools, like things that we would need to do our job, like we did not have bikes and had to walk everywhere … but we always find a solution, so at the end, there is no problem…” (GPC1/6.)*

The majority of health mediators expressed their wish to be employed six hours per day or full-time since they found their wages in half-time employment too low.

*“Full time [work] would be much better because the money would be higher, and we could fill the hours working because we could do any tasks they [the supervisor] give us.” (GPC2/9.)*

*“I would love to keep doing this but, let’s admit, the money is slim.” (GPC4/11.)*

### Theme 3: Future prospects

#### Future of the Model Programme and suggestion for improvement

Regarding the continuation of the Programme, 90% of the mediators responded that they would like to see the Programme continue, including the work of health mediators.

*“I believe we can improve this more, take this further, we can think of more things to add to it, we can expand it, I think there are a lot of opportunities in this.” (GPC1/4.)*

*“I absolutely hope so. I think there’s a big need for this, our work also says that it would be important to have an occupation like this running for longer, either under this name or some other name, but there’s definitely reason for this to exist.” (GPC1/6.)*

27 of them said they hoped the Programme including their work would continue:

*“I don’t have such big plans, really, but I would definitely like to continue doing this work, I would like to continue doing it successfully, to the best of my abilities, I am open to any training or any new work, new areas also interest me, but I think I like this job and I would like to continue doing it.” (GPC1/6.)*

In terms of improvement, they suggested to have more community health promotion Programmes; at-home screenings for the elderly; more specialist doctors such as dermatologist, rheumatologist; and a wider range of health assessment procedures including blood tests, abdominal ultrasound, screening for cervical cancer and mammography (the two latter were not part of health status assessment because these are organized according to a national protocol), and improved external communication, that is, greater publicity for the Programme.

*“More advertisement on TV maybe, in newspapers, on the news, on radio, maybe flyers.” (GPC2/4.)*

“I was in O*, in the hospital for an examination [escorting a patient], and the Head Physician asked, well, what do I need the blood test results for? Why did I need to have it done? And I told him, but he hadn’t heard about it [the Programme]. And I told him, and I said this is great, well they could also have it, it would be good here in O*. And, and he was curious, but we need bigger publicity. We need to advertise [the Programme] more.” (GPC2/4.)

They also suggested more training for themselves, particularly improving communication skills; training in informal team building for non-medical workers, and they suggested further on-the-job vocational training:

*“I would like to have [another] vocational training in the Programme, let’s say social assistant…” (GPC2/1.)*

#### Personal plans for the future

Two-third of the interviewees (25 persons) had been unemployed before the Programme, and twenty-two of them obtained on-the-job vocational qualification in the framework of the Programme. Therefore, one of the items in the structured interview related to their plans to continue to work in healthcare after the end of the Programme (scheduled for June 2016 at the time of the interviews that was later extended to May 2017). One did not want to continue, and three were uncertain, but the overwhelming majority of the mediators (36 persons) expressed their desire to work either in this position or some similar position in healthcare. They also expressed their willingness to learn new skills or vocation.

*“I don’t have such big plans, but I would like to do this work in the future, to work successfully…I am fully open for anything, new training or new work, I’m interested in new fields but I like this work and would do it if I can…” (GPC1/6.)*

*“I would add that I would like [the Programme] to continue. It has been almost three years that I am working here and would like to keep doing it. After all, I signed up because I was interested. So I would like to do this or something similar in healthcare.” (GPC4/3.)*

## Discussion

Health mediators of the Primary Care Model Programme of Hungary had positive experiences and opinions about the Programme and their work. This was reflected by the fact that all of them consented to be interviewed and readily answered all questions, not hiding their occasional criticisms. They considered their insider knowledge of Roma communities as an advantage in their work as health mediators, and they were overwhelmingly in support of keeping such a position in primary health care. Becoming member of the primary health care team, on-the job training, increase in self-esteem and increased respect in the community and in their families as well as becoming more health-conscious were mentioned favourably by the majority of the interviewees. Critical remarks were mostly related to the slowness of procurement of tools and devices (for example, tablets and bikes for them) that was due to the mandatory but very slow process of public procurement, and tardiness of some administrative issues that was due to the fact that health mediators were employed by the management of the Programme in the capital so most administrative matters had to be taken care of centrally and not in the GP clusters. Altogether, health mediators were overwhelmingly in favour of their positions and work arrangements, and made a number of useful suggestions for the future.

The applied method of structured interviews is one of the strengths of this research because it revealed insights and experiences that no pre-designed questionnaire or external observer could uncover. Among further advantages are the fact that all mediators consented to be interviewed; saturation was reached, and all interviewers established good rapport with their interviewees. Qualitative methods can substantially inform and improve health care decisions but not favoured and therefore underutilized by certain scientific journals [21].

However, some limitations must also be mentioned such as the fact that mediators who quit their jobs before the start of this research were not interviewed. Considering the full turnover of health mediators between the beginning of the operation of the Programme (July 2013) and the start of the interviews (February 2016), 17 health mediators were not interviewed who had been employed for any length in the Programme but left before interviews were conducted. Based on informal discussions, most of them were not satisfied with either the type or the schedule of the work, and/or the income it provided.

Other workers of the Programme including supervisors of the mediators, as well as patients and family members were not interviewed but we do not consider this a limitation since their views would have provided a quite different perspective compared to that of health mediators. It has been revealed in other qualitative studies that even health professionals in different facilities [22] or in different positions [23] have varying perceptions about aspects of primary care such as barriers to patient access and care or professional collaboration, not to mention non-professional workers including health mediators.

Health mediators can be considered special community health workers [24] who have worked in Central and Eastern European countries [12] to help address the dismal health conditions of Roma population groups that are compounded by numerous barriers to access and use of health services [25]. Access of disadvantaged Roma to health services may be hindered by a number of factors of which registering with services, cultural differences, experience of discrimination, the vulnerable population’s lack of education and health literacy, psychological barriers and economic barriers were found to be the most frequent ones [23]. Problems of access to health care among Roma can be significantly ameliorated by perceived social support provided by family members and friends [26] but this may not be sufficient to overcome the system of complex and interrelated local barriers to health care among those who live in segregated conditions [27]. Roma persons, typically women, recruited from local communities, willing and able to take up the role and getting training in health mediation can provide the type of social support that overcomes any potential obstacles by negotiation and perseverance [12]. This earlier conclusion was amply supported by our own research. True to the original Latin word [28], health mediators stand in the middle becoming agents of intervention, and true to the spirit of the EU, they embody the contemporary bridges on euro notes [29] reminding us all that just as riverbanks are to be connected so should those in need be connected with those who are trained and able to help. This connecting function has been and will be well served by health (or intercultural) mediators [30] who should become permanent employees in institutionalized positions in the human resources of primary health care, particularly in areas with sizable Roma populations.

## Conclusions

The Hungarian Primary Care Model Programme that operated between 2013–2017 in Hungary was globally the first in which health mediators, non-professional workers became equal members of the primary care team as salaried employees. Interviews with 40 health mediators of the Programme provided ample evidence that they felt they not only fulfilled their major tasks and improved access of Roma and non-Roma disadvantaged groups to primary health care services but their own attitudes to health and their social status also increased. They felt as useful members of the primary care teams (‘GP clusters’) established in the Programme. Their overwhelmingly positive experiences, along with their useful insights for improvement – notwithstanding some criticism – strengthens the argument for the institutionalization of health mediators by establishing permanent positions for them in primary care especially in areas with large numbers of disadvantaged Roma populations.

## Data Availability

Data collected in the framework of the Programme are managed by the National Public Health Institute of Hungary. Transcripts are available on reasonable request from the first author after written permission is issued by the director of the National Public Health Institute.

## Appendix

### The interview questions

1. Why did you join the Programme? Please mention at least three measurable incentive factor which compelled you to undertake this work.
2. How did your life change since you have worked as an assistant health mediator? How do you see yourself now in comparison with period before the Programme? *(self-esteem, self-image, individual development)*
3. How did starting to work and the job of assistant health mediator change your status in the family and the local community? *(support/pulling back)*
4. Do you think that ethnic origin was a factor in who was hired as an assistant health mediator? What ethnic origin do you consider yourself to have? In your opinion, does ethnic origin have any significance in healthcare?
5. On the basis of the current situation do you think that this work will provide a job opportunity in the future? What are your plans for the period after June 2016 (the planned end of the Programme that was later extended to May 2017)?
6. What were your most characteristic conflicts with the clients, patients? How do you resolve these?
7. What situations cause stress for you? What feelings do you have in such situations? How do you cope with situations that cause you stress? (*Would require help for yourself, do you have someone to discuss your conflicts with*?)
8. How important do you think setting a good example is from the point of view of health consciousness? Are you setting a good example? Are your colleagues in the practice cluster setting a good example? (*harmful addictions … concretely ask about smoking.*)
9. What do you consider the most important part of your work and what is the most important result of your work? If you had to argue for the need for assistant health mediators in primary care, what arguments would mention? *(Are you proud to be a part of the system and of working alongside the doctors? Mention 2–3 concrete examples of the most important results of your work.)*
10. What do you see as the strengths and the weaknesses of the Programme? (*How could the Programme be made more efficient and effective?*)
11. How would you characterise your relationship with the public health coordinator? Do you receive from them all the assistance you need for your everyday tasks? How would you characterise your connection with the practice cluster coordinator? Do you receive from them help for the performance of your work? How would you characterise your relationship with your employer in Budapest (i.e. those working in the headquarters in Budapest)?
12. What do you think about their work? Do they provide all assistance 1) for the performance of your work? 2) for the proper operation of the practice cluster? *(flow of information, tools, materials, equipment, *etc*.)*
13. Is there any issue or comment in connection with your work that you would consider important to share, but was not addressed in the above questions?

## Abbreviations

EU: European Union
RHM: Roma health mediators
GP: General practitioner
GPC: General Practitioners’ practice cluster
